# Experiences of a “COVID protected” robotic surgical centre for colorectal and urological cancer in the COVID-19 pandemic

**DOI:** 10.1007/s11701-021-01199-3

**Published:** 2021-02-11

**Authors:** Jeremy R. Huddy, Matthew Crockett, A Shiyam Nizar, Ralph Smith, Manar Malki, Neil Barber, Henry S. Tilney

**Affiliations:** 1 Department of Colorectal Surgery, Frimley Park Hospital, Camberley, UK; 2 Frimley Renal Cancer Centre, Frimley Park Hospital, Camberley, UK; 3 Department of Surgery & Cancer, Imperial College London, London, UK

**Keywords:** Coronavirus, Colorectal surgery, Urology

## Abstract

The recent COVID-19 pandemic led to the cancellation of elective surgery across the United Kingdom. Re-establishing elective surgery in a manner that ensures patient and staff safety has been a priority. We report our experience and patient outcomes from setting up a “COVID protected” robotic unit for colorectal and renal surgery that housed both the da Vinci Si (Intuitive, Sunnyvale, CA, USA) and the Versius (CMR Surgical, Cambridge, UK) robotic systems. “COVID protected” robotic surgery was undertaken in a day-surgical unit attached to the main hospital. A standard operating procedure was developed in collaboration with the trust COVID-19 leadership team and adapted to national recommendations. 60 patients underwent elective robotic surgery in the initial 10-weeks of the study. This included 10 colorectal procedures and 50 urology procedures. Median length of stay was 4 days for rectal cancer procedures, 2 days less than prior to the COVID period, and 1 day for renal procedures. There were no instances of in-patient coronavirus transmission. Six rectal cancer patients waited more than 62 days for their surgery because of the initial COVID peak but none had an increase T-stage between pre-operative staging and post-operative histology. Robotic surgery can be undertaken in “COVID protected” units within acute hospitals in a safe way that mitigates the increased risk of undergoing major surgery in the current pandemic. Some benefits were seen such as reduced length of stay for colorectal patients that may be associated with having a dedicated unit for elective robotic surgical services.

## Introduction

Preparation for the current coronavirus pandemic resulted in the cessation of elective surgery at NHS hospitals with an estimated 43,000 operations being cancelled per week in the United Kingdom alone [1]. This freed up intensive care beds and the anaesthetic workforce to manage patients admitted with coronavirus [2]. Minimally invasive surgical techniques such as robotics have been particularly affected by the associated changes to surgical practice as early recommendations from national bodies favoured open approaches to surgery largely based on the theoretical risk of aerosolisation of virus particles in carbon dioxide insufflation [3]. However, these guidelines have been relaxed over recent weeks as we shift focus to the resumption of elective services in a way that mitigates the risks for patients and staff.

The risk of mortality from contracting coronavirus peri-operatively has been estimated at 21–24% [4, 5]. Risk factors for adverse outcomes in patients developing COVID-19 include male sex, diabetes, cardiac co-morbidity and cancer [6, 7]. Ethnic minority groups have also been reported to be disproportionately affected by COVID-19 with both a higher incidence and severity [8].

Robotic surgery presented unique challenges during the pandemic. Surgeons may need to compromise their recommended personal protective equipment to engage with robot consoles, operating time may be increased compared to open or laparoscopic approaches and there may be a need for dedicated theatres with appropriately trained staff at a time when resources are already stretched. However, robotic surgery may also present advantages to patients in the current pandemic by reducing length of stay thereby freeing up hospital beds and reducing the risk of virus transmission during post-operative recovery [9], reducing pulmonary stresses with lower pneumoperitoneum pressures [10] and minimising personnel required to be present in the direct vicinity of the patient during surgery [11].

Uncertainty exists as to the best way to safely re-introduce elective surgical services in this period of increased risk. However, a failure to do this will inevitably lead to a growing cohort of patients who experience adverse outcomes from COVID-19, not through the virus itself but as a result of delays to, or compromises in treatment, and potential progression of, other medical conditions such as cancer [12]. Considerations for best practice have been proposed [13] and guidelines published from national bodies including the Association of Coloproctology of Great Britain and Ireland (ACPGBI) [14], the British Association of Urological Surgeons (BAUS) [15] and the Royal College of Surgeons of England [16] to safely re-introduce elective surgery. These recommendations include establishing cold operating sites, screening of staff and patients and responding to fluctuating resource availability based on local environmental factors [16]. Alternative surgical strategies, such as stoma formation, intended to reduce morbidity and length of stay may be recommended [17]. However, to date there has been a paucity of correspondence regarding the role of robotic surgery in the current climate.

We report our early experience of establishing a “COVID protected” unit for elective robotic surgery at a large acute care district general hospital with an established robotic surgery programme and a tertiary referral service for renal cancer.

## Methods

A “COVID protected” site for major elective surgery, including all robotic procedures, at Frimley Park Hospital was established in a purpose-built day surgery unit [18]. In summary, a standard operating policy was designed to manage priority two and three cases [19] in a protected area, while acute services continued in the hospital. This unit was attached to the main hospital building but had a separate entrance for patients and staff. The unit had 16 individual patient bays (initially reduced to 10 to enhance social distancing measures between patients, but subsequently returned to 16 as infection control measures were relaxed) and a theatre recovery staffed for 4 patients. Two robotic operating theatres were adjacent to the ward; one housed the da Vinci Si robotic system (Intuitive, Sunnyvale, CA, USA) and the other the Versius (CMR Surgical, Cambridge, UK) system. Some minor structural changes were required before the unit was used including removing sliding doors from individual patient cubicles to accommodate in-patient beds and provide more space should a resuscitation team be required.

All staff working in the unit isolated for at least 48 h prior to their first shift, were excluded from the main hospital building and had weekly drive through swab testing for coronavirus RNA. Staff were asked to minimise contact with likely COVID patients for 7 days before entering the unit. All patients were asked to self-isolate for 14 days prior to their surgery and had both a coronavirus RNA swab. In the first 2 weeks, a chest CT was performed within 48 h of admission although the requirement for chest CT was removed soon after initiation of the pathway, in line with national guidance [20]. Nursing staff and theatre staff with experience in robotic surgery were seconded from the relevant surgical wards and supported by staff from a local private healthcare provider. A full-time stoma nurse was also based on the ward.

Patients were prioritised according to national criteria and vulnerability scores were recorded [19]. Pre-assessment was undertaken by telephone initially with appointments made at non-acute sites for patients who required investigations such as phlebotomy. Operating lists were reduced to avoid pressure on the limited number of beds, and to account for extended theatre protocols to mitigate COVID-risk. For example, an all day list for renal cancer would be two robotic procedures rather than the usual practice of three.

Indications for robotic surgery were unchanged during the pandemic. Rectal cancer surgery was started laparoscopically with the da Vinci Si system (Intuitive, Sunnyvale, CA, USA) utilised for the total mesenteric excision (TME) dissection. The Versius robot (CMR Surgical, Cambridge, UK) surgical program had been recently launched at Frimley prior to COVID but was paused prior to the initial peak. It was restarted with selected colonic resections in July 2020 when patients deferred during the initial coronavirus peak had received their surgery. Patients undergoing colorectal resection received pre-operative bowel preparation and oral antibiotics at home in keeping with local policy. Pyeloplasty, nephro-ureterectomy, partial and some radical nephrectomies were performed on the same da Vinci system. All procedures were performed using the Airseal insufflator (ConMed, Utica, NY, USA), which has been suggested may reduce the risk of aerosolization of the virus due to lower pneumoperitoneal pressures and the use of an ultralow particulate air filter (rated to 0.1 µm) [21]. All cases were initially undertaken with two consultant surgeons in attendance and a consultant anaesthetist, according to initial guidelines [17]. Although these recommendations were later relaxed, rectal cancers are still considered a ‘two consultant’ procedure, and the requirements for separate 24 h, clean, post-operative cover meant that the presence of more than one clean specialist was sensible.

Recovery staff were trained in the management of epidural catheters, rectus sheath catheters and arterial lines. This meant that some patients who required a higher level of post-operative support, in the case of conversions to open surgery or other issues, could remain in recovery for up to 48 h, with two recovery beds identified as high dependency areas. An established enhanced recovery protocol was used for the post-operative management of patients. Daily, subspecialty, consultant led ward rounds were undertaken of all patients. Personal protective equipment was used for every patient interaction with visors and filtering facepiece respirators used for aerosol generating procedures including surgery, with the exception of robotic console-time, during which the use of a visor was not possible. Pathways were in place for the safe transfer of patients to the main hospital if cross-sectional imaging was required. Full access was available to the hospital clinical laboratory services including blood bank and the major haemorrhage pathway if required. Supplies from the main hospital including patient food, blood products and theatre supplies were handed over in an “airlock” corridor from a main hospital porter to a “COVID protected” unit porter, both in personal protective equipment, and containers were cleaned before being brought into the unit.

A prospective database of all patients admitted to the unit was maintained. Patients were also asked to complete a trust in-patient survey on discharge. Patient feedback regarding the unit has been previously reported [18]. Results are reported using descriptive statistics.

## Results

Robotic elective surgery was re-established on the 12th May 2020. At this time, there were 69 inpatients with confirmed coronavirus (17 in critical-care areas) at Frimley Park Hospital, down from a peak of 186.

### Patient outcomes

60 patients underwent elective robotic surgery in the unit between 12th May and 30th July 2020. This included 10 colorectal procedures and 50 urology procedures. One other colorectal patient scheduled for robotic low anterior resection had had previous mesh rectopexy and, therefore, the operation was converted to an open procedure prior to docking the robot. This patient has not been included in the analysis. Patient demographics and outcomes are presented in Tables 1, 2. All patients who had low anterior resections for rectal cancer had a defunctioning ileostomy fashioned. No patients tested positive for coronavirus RNA or demonstrated features of coronavirus in their pre-operative investigation. Two staff members tested positive in their routine weekly swab screening including one surgeon.

**Table 1:** Patient demographics and outcomes for patients undergoing robotic colorectal procedures

	*n* (%) (*n* = 10)
Male	5 (50)
Age (years) median (IQR)	77 (59–79)
P score [19]	P3 10 (100)
V score [19]	V1 4 (40) V2 6 (60)
Operation	Da Vinci robot cases: Low Anterior Resection 6 (60) APER 2 (20) Versius robot cases: Sigmoid Colectomy 1 (10) High Anterior Resection 1 (10)
Operating time median (minutes) (IQR)	264 (279–324)
Length of stay (days) median (IQR)	4 (4–4.75)
Pre-operative staging	
T-staging	
T1	1 (10)
T2	2 (20)
T3	7 (70)
Lymph node involvement	
N0	3 (30)
N1	5 (50)
N2	2 (20)
Post-operative pathology	
T-staging	
Tubulovillous adenoma	1 (10)
T1	1 (10)
T2	3 (30)
T3	5 (50)
Lymph nodes resected median (IQR)	23 (18–30)
Lymph node involvement	
N0	7 (70)
N1	3 (30)
Extramural vascular invasion	2 (20)
Complications	Ileus requiring TPN 1 (10)
Readmissions	0 (0)

*APER* abdomino-perineal excision of the rectum

**Table 2:** Patient demographics and outcomes for patients undergoing robotic urological procedures

Variable	Value
Total	50
Age, median (IQR)	67 (57–76)
Male: female (%)	33:17 (66%:34%)
Left: right (%)	19:31 (38%:62%)
Operation, *n*	50
RAPN, *n* (%)	39 (78%)
RARN, *n* (%)	4 (8%)
RNU, *n* (%)	3 (6%)
RAP, *n* (%)	3 (6%)
Robotic Adrenalectomy, *n* (%)	1 (2%)
Operation Time, mins, median (IQR)	135 (95–162.5)
Estimated Blood Loss, ml, median (IQR)	50 (10–100)
Length of Stay, median (IQR)	1 (1–2)
RAPN tumour size	30 (25–38)
RAPN stage	
1a	25 (80%)
1b	5 (16%)
2a	1 (4%)
RAPN histology	Malignant (31, 79%) Benign (8, 21%)
Complications^a^	
Intraoperative	1–tumour rupture
Post-operative	1–ileus 3–transfusion
Readmissions	0

*RAPN* robotic assisted partial nephrectomy, *RARN* robotic assisted partial nephrectomy, *RNU* robotic assisted nephro-ureterectomy, *RAP* robotic assisted pyeloplasty

^a^All 3 post-operative complications occurred in patients receiving RNU

There were no known instances of patients included in this study developing coronavirus during their peri-operative in-patient stay. No patient required attendance from the hospital medical emergency team. Two patients required transfer to the main hospital for cross-sectional imaging. One patient required a planned transfer to the hospital intensive care facilities for post-operative care.

The first six patients with rectal cancer had their surgery deferred because of the initial coronavirus peak and, therefore, breached the 62-day treatment target. Median time from diagnosis to treatment for colorectal patients (excluding one patient who underwent neoadjuvant chemoradiotherapy) was 94 days (inter-quartile range 51–105). No patient with colorectal cancer had an increase in the T-stage from pre-operative staging to post-operative histology. Three (30%) of patients were downstaged post-operatively in respect to their T-stage. Two (20%) patients who were initially staged as N0 pre-operatively had lymph node involvement in their post-operative histology (one N1a and one N1b). Six patients (60%) were downstaged post-operatively in respect to their N-stage.

## Discussion

We report our experience of re-establishing elective robotic surgery immediately following the initial surge period of the coronavirus outbreak. We believe this to be the first robotic unit in the world to house both the da Vinci Si (Intuitive, Sunnyvale, CA, USA) and Versius (CMR Surgical, Cambridge, UK) robotic systems in adjacent theatres.

Median length of stay for robotic rectal cancer procedures was 4 days, both Versius cases (one sigmoid cancer and one upper rectal cancer) were discharged on day 2 without stomas and no post-operative complications. This compares to a median length of stay of 6 days for patients having surgery for rectal cancer before the coronavirus pandemic. The potential reduction in in-patient length of stay may be explained by their post-operative management being undertaken in a dedicated elective surgical department, with relatively high nurse to patient ratios and high levels of consultant input, where staff were able to focus on peri-operative care needs and enhanced recovery without the traditional ward mix of elective and emergency patients. Having a stoma nurse based on the unit allowed stoma competency to be achieved early in the post-operative recovery, although visiting restrictions meant that family could not be directly involved in education sessions.

During the initial coronavirus peak colorectal cancer operations were deferred, with patients monitored, in our trust rather than proceeding in this period of increased risk when guidelines recommended compromises to standard practice including open surgery and the routine defunctioning of patients rather than primary anastomosis [17]. The results justify this approach as no patients had a higher T-stage tumour than had been demonstrated in their imaging at diagnosis, conversely 30% of patients were downstaged. Lymph node status was more variable but has previously been demonstrated to offer little prognostic value [22].

For urology procedures, all measured outcomes including length of stay were unchanged from before the pandemic. All post-operative complications, including both post-operative CT scans, occurred in patients receiving a robotic nephro-ureterectomy. We accommodated the losses in efficiency, such as operating in full personal protective equipment, full air change, and operating in a new environment by reducing capacity (from three to two cases). This helped create a relaxed environment and even provided additional training opportunities for the fellow in appropriate cases.

Patient satisfaction on the unit was high, demonstrated by the in-patient survey data collected on patient discharge [18]. It was reassuring that patients felt confident undergoing major surgical procedures in hospital at a time of increased risk, particularly given the number of patients with cancer and comorbidities which put them into high risk categories for adverse outcomes should they have developed the respiratory complications of coronavirus. Patient’s relatives were not allowed to visit the ward and efforts were made to keep family members informed of their relatives progress, such as phoning relatives at the end of operations, with the patient’s consent, which forms part of our normal practice in any event.

Pathways for patients were established in advance of recommencing elective surgery, particularly in respect to the management of the deteriorating patient. This balanced patient safety against the desire to keep patients and staff isolated from the main hospital building. Provisions were not in place to run a COVID protected operating theatre out of hours. However, a patient unwell enough to be returned to theatre out of hours was felt to be likely to require intensive care in the post-operative period anyway and, therefore, transfer to the non-COVID facilities in the main hospital would have been appropriate if this circumstance had arisen. Following discharge patients could not be re-admitted to the ward. The unit protocols followed, and responded to, the latest guidelines from national bodies. An example of this evolving practice was that towards the end of the study period pre-operative CT chest imaging was no longer recommended for all patients.

Staffing was a significant challenge in performing major surgery in a separate unit. Twenty-four hour medical cover was necessary, with all doctors required to isolate from the main hospital prior to working their shifts. This led to an increase in the number of night shifts being undertaken by junior doctors but fortunately this came at a time when staff were returning to general surgery after redeployment to critical care in the surge period. Consultant surgical staff were required to remain “clean” and cover out of hours in the unit in addition to the routine emergency on-call rota. This highlights the importance of the advice from the Royal College of Surgeons of England that elective surgery services should only be recommenced in an environment with demonstrated reduction in new cases of coronavirus [16]. One surgeon tested positive for coronavirus during the study period. It is not possible to ascertain if this was contracted in the unit or other environments. However, although some components of personal protective equipment were not feasible at the robotic console such as face masks, we believe the judicious use of staff and patient testing meant that overall risks were low.

Anaesthetic staff being able to appropriately separate from COVID patients prior to attendance on the unit was initially a concern. When deciding on the location of a clean site, the senior trust team had to account for numerous risks and attempt to mitigate them. Options included the use of independent sector providers and a short-stay treatment facility. The proximity to acute services, if necessary, and the fact that there were insufficient critical-care staff (medical and nursing) to institute an off-site “mini-Intensive care unit” led the conclusion that an isolated bubble within a contaminated hospital” was the best option for our individual circumstances. As the burden of COVID-related illness in the main hospital reduced, it was possible to create a cohort of “clean” anaesthetists working solely in the COVID protected elective site. This model allowed for 24-h anaesthetic cover available for advice to the junior teams regarding cardiorespiratory issues and pain management post-operatively.

As expected, when designing a new standard operating procedure, there were some initial difficulties. At first all patients were required to attend three pre-operative appointments at different hospitals (one for a CT chest whilst this was recommended, one for pre-operative phlebotomy and urine culture, and one for a swab) before being admitted to the COVID protected site. One of our patients had severe mobility difficulties requiring hospital transport that made the attendance at all the appointments logistically impossible.

Working in a contained unit without time pressure and having a recovery area and ward opposite the operating theatres proved to be efficient and staff morale was high. The unit is expected to continue to run in this fashion, with regular review from the trust’s emergency incident command structure, before surgery can return to the hospital’s main theatre complex where currently only emergency surgery and obstetrics are based.

This study has limitations. Patient satisfaction data were collected for all patients having surgery in the unit. However, the unit was also used by patients having major open and laparoscopic surgery under several specialties. Therefore, it is not possible to report data for the robotic patients in isolation. Whilst there are no known cases of coronavirus transmission the majority of patients did not have routine swabbing after admission (this was only undertaken if patients showed symptoms or if they were still an in-patient at 7 days). Consequently, there may have been asymptomatic transmission that we did not detect.

In summary, we present the early outcomes of a rapidly instituted facility designed to provide urgent robotic surgery, using two different robotic systems, in as safe a manner as possible in the context of the early recovery period following the first peak of local COVID infection. The model has the ability to flex in response to secondary peaks of infection, and in the event of further “surges” it can be suspended and rapidly re-implemented. Levels of morbidity have been low and patient satisfaction high. Early results suggest that benefits to this model of service delivery might include shortened lengths of stay.

## Data Availability

Not applicable.
